# Comparable postoperative outcomes in patients treated with either open or arthroscopic trochleoplasty for patella dysplasia

**DOI:** 10.1186/s43019-024-00247-0

**Published:** 2024-12-02

**Authors:** Georg Riedl, Lukas A. Holzer, Vinzenz Smekal

**Affiliations:** 1 Department of Orthopaedics Surgery, AUVA Trauma Center Klagenfurt, Waidmannsdorferstraße 35, 9020 Klagenfurt Am Wörthersee, Austria; 2 Department of Orthopaedics and Trauma Surgery, Innsbruck Medical University, Innsbruck, Austria; 3 Sonderkrankenanstalt für Orthopädie Warmbad-Villach, Villach, Austria

**Keywords:** Patella instability, Patella dysplasia, Trochleoplasty, Arthroscopic technique

## Abstract

**Background:**

The aim of this study was to compare the clinical and radiologic results of the arthroscopic and the open trochleoplasty techniques.

**Methods:**

A total of 83 trochleoplasties in 83 patients were performed between 2014 and 2021 in one institution. Surgical indications for trochleoplasty were recurrent patellofemoral instability and a lateral trochlear inclination angle (LTI) ≤ 11˚ and a trochlear depth ≤ 6 mm. Of the trochleoplasties, 40 were done by open technique (OT) and 43 by arthroscopic technique (AT). In every case an additional medial patellofemoral ligament (MPFL) reconstruction was performed. Additional tuberosity tibia transfer and/or de-rotation of the femur were done when indicated. Pre- and postoperative magnetic resonance imaging (MRI) were analyzed in respect to LTI, trochlear depth, and lateralization of the patella. Postoperative subjective clinical assessment was done using the Kujala Score, Banff II Score, Tegner Score, and Marx Score.

**Results:**

Of the patients, 15 with OT and 20 with AT were available for follow-up. The mean follow-up was 29.9 months in the OT group and 12.7 months in the AT group. No re-dislocation was observed in either groups. A significant reduction of LTI, increase of trochlear depth, and a reduction of lateralization of the patella was observed between the pre- and postoperative MRI scans in both groups. No significant difference in the observed MRI parameters was found between the two groups. Neither was there a difference in the postoperative Kujala Score, Banff II Score, Tegner Score, and Marx Score between the two groups. Length of stay was on average 6.2 days in the AT group and 8.1 days in the OT group. The surgical time was on average 141 min in the OT group and 160 min in the AT group.

**Conclusion:**

We found that patients undergoing an arthroscopic trochleoplasty had a comparable outcome with respect to clinical and radiological parameters compared with patients treated by open trochleoplasty.

## Background

Trochleoplasty is a well-established and generally accepted treatment for patients with severe trochlear dysplasia associated with persistent patellar instability [1].

Recent clinical studies as well as biomechanical studies demonstrate the relevance of a dysplastic trochlea for patella instability and the clinical aspect [2–4]. The results indicate a decrease of the lateral maltracking and reducing of the risk of re-dislocation [5]. Various surgical techniques have been developed for the treatment of patella instability, such as the deepening trochleoplasty [6].

The deepening trochleoplasty is a reliable and safe procedure [7]. Nevertheless, the open technique causes a excessive soft-tissue damage due to an extensive approach and prolonged rehabilitation. In a case series, Carstensen et al. reported of an incidence of 27% arthrofibrosis of patients who had open trochleoplasty [8]. The high rate of arthrofibrosis was considered as a consequence of surgical trauma. To minimize soft tissue trauma, an arthroscopic technique (AT) was established, which was based on a technique that was described previously [9, 10].

There are numerous studies about the open techniques (OT), but only a few reports about the arthroscopic technique, and no studies comparing open and arthroscopic trochleoplasties [7, 9–14].

The aim of the study was to compare arthroscopic and open trochleoplasty in respect to radiological and clinical outcomes. We hypothesized that the radiological and clinical outcomes would be similar between the arthroscopic and open trochleoplasty.

## Methods

### Patients

This was a retrospective cohort study with prospectively collected follow-up data that included patients who underwent either arthroscopic or open trochleoplasty in combination with medial patellofemoral ligament (MPFL) reconstruction with the gracilis or quadriceps tendon. The surgical interventions were done by two board certified surgeons (G.R. and V.S.) in our hospital between April 2014 and May 2021. Surgical indications for trochleoplasty were patella instability with recurrent patellar dislocation (minimum of two events or one event with contralateral recurrent dislocations) and presence of trochlear dysplasia without previous surgery of patella dislocation. Exclusion criteria were a missing pre- or postoperative magnetic resonance imaging (MRI), previous surgical interventions at the patellofemoral joint, osteoarthritis Kellgren–Lawrence score higher than Grade 2, and a follow-up of less than 11 months [15].

### Surgical techniques

Open trochleoplasty—“Bereiter technique” [16]

The OT was the standard procedure in our hospital until 2018; since then, the AT has been performed routinely for the treatment of trochlea dysplasia.

The patient is in a supine position under general or spinal anesthesia. A tourniquet is applied. Parapatellar lateral arthrotomy with retraction of the patella medially is carried out. The articular cartilage in the proximal aspect of the trochlear cartilage is separated from the synovium. An osteochondral flap is raised with curved chisels approximately 5 mm cranial of the notch. A new trochlear groove is deepened using different chisels and a burr. The thinned out osteochondral flap is then molded into the newly formed groove and fixed with three bone anchors and a Vicryl tape suture. After every trochleoplasty, a MPFL reconstruction is done with a quadriceps or gracilis tendon [17].

Arthroscopic trochleoplasty—“Lars Blønd technique” [10, 13]

The patient is in a supine position under general anesthesia. We start the operation without a tourniquet; therefore, the systolic blood pressure should be constantly near 90 mmHg (healthy young people normally tolerate this blood pressure without problems). That is why we recommend the operation under general anesthesia. In addition to the standard portal, we need to have in hand a proximal lateral working portal and a proximal medial portal for the arthroscope. The first step is to remove the fatty tissue of the bump with the bipolar radiofrequency instrument (Bipolar RF) instrument and start to cut off the subchondral bone with a 4 mm burr. With the power rasp, additional bone is thinned out to make the trochlea flap bendable. Bony bridges on the medial and lateral side are detached with a chisel. At the end, a bone anchor with a Vicryl tape suture is inserted from the anteromedial or anterolateral portal (depending on the accessibility) near the notch, and the tape is stretched over the shield to proximal lateral and central, fixed with two additional bone anchors. The MPFL reconstruction (gracilis tendon) is done with a sterile tourniquet that is applied after the trochleoplasty have been performed.

If indicated, a distalization of the patella is performed as a last step after either AT or OT.

In the case of an coronal plane alignment correction or de-rotational femoral osteotomy, an open technique is done as a standard.

### Second-look surgery

In pediatric or adolescent patients, removal of metal hardware in combination with a diagnostic knee scope was offered 1 year after index surgery.

### Clinical assessment

The clinical assessment included a standard knee examination for exclusion of ligamentous instabilty not related to the patellofemoral joint. Specific patellofemoral examination included the j-sign, malrotation, overall leg alignment, apprehension sign, and the grade of instability evaluated by glide test [18].

### Radiological assessment

Radiological evaluation of all included patients included pre- and postoperative x-rays in two planes, MRI scan, and leg alignment x-ray. For selected cases in patients with a positive apprehension test above 60˚ and clinical suspicion of rotational malalignment, a rotational profile by computed tomography (CT) or MRI of hip, knee, and ankle was done.

The patellofemoral joint including the trochlea were evaluated by MRI before and after intervention. The MRI was done using a 1.5 Tesla Phillips within the postoperative examination. The pre- and postoperative MRI evaluation was done by the first author (G.R.).

**Fig. 1:**
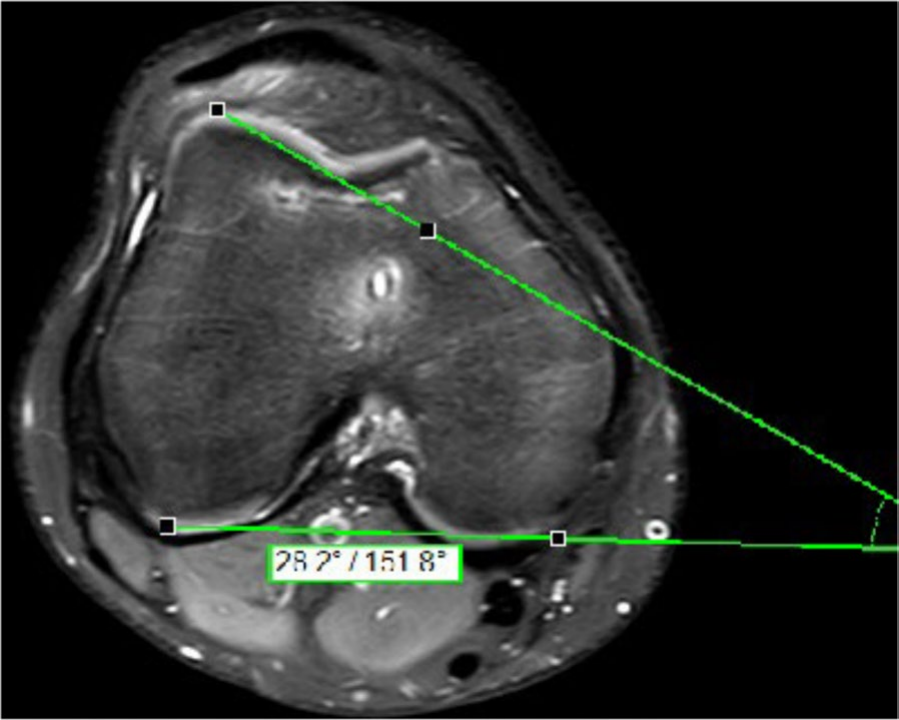
Measurement of the lateral trochlea inclination (LTI): The LTI is measured in the MRI slices 3 cm above the femorotibial joint space by measuring the angle between a line tangential to the subchondral bone of the posterior aspect of the femoral condyles and a line tangential to the subchondral bone of the lateral trochlear facet

**Fig. 2:**
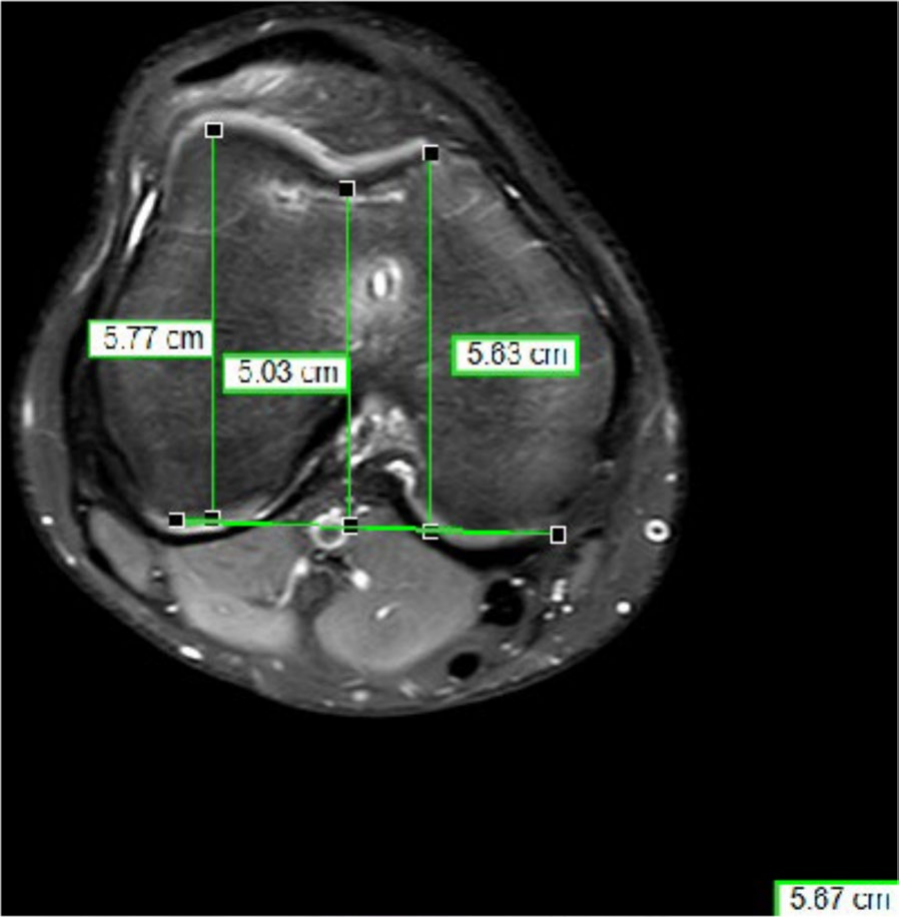
Measurement of the trochlear depth: The trochlea depth is measured in the MRI slices 3 cm above the femorotibial joint space by measuring the maximal anteroposterior distance of the medial (distance **a**) and lateral femoral (distance **b**) condyle and the minimal anteroposterior distance between the deepest point of the trochlear groove and the line paralleling the posterior outlines of the femoral condyles (distance **c**). Trochlear depth was calculated by the formula ([a + b]/2) − c

**Fig. 3:**
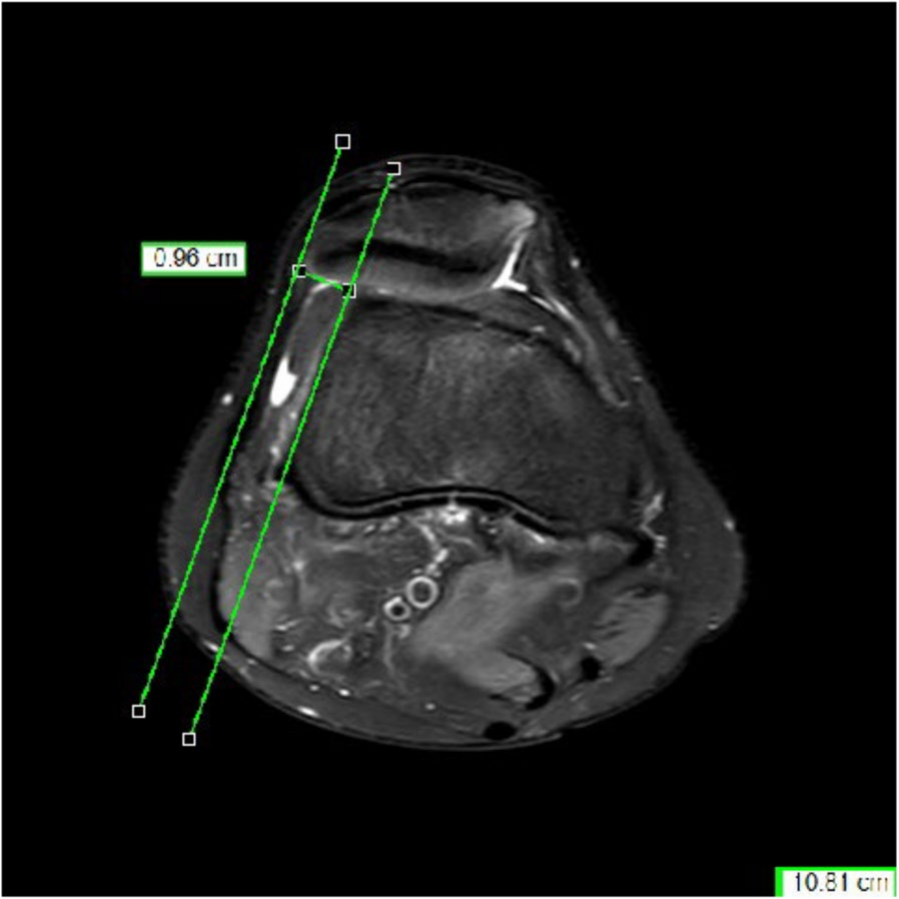
Measurement of the lateralization of patella: Lateralization is the distance of the line paralleling the lateral margin of the lateral condyle to the most lateral point of the patella

The measure was done on axial MRI at the most proximal level with the posterior condylar line as reference and with lateral trochlea inclination (LTI) < 11˚ (Fig. 1) and a trochlear depth < 3 mm (Fig. 2). Evaluation of lateralization of the patella was carried out according to Pfirmann > 6 mm (Fig. 3) [19].

The lateral trochlea inclination is measured in the MRI slices 3 cm above the femorotibial joint space by measuring the angle between a line tangential to the subchondral bone of the posterior aspect of the femoral condyles and a line tangential to the subchondral bone of the lateral trochlear facet [20]. In the same slices, the trochlear groove can be measured by measuring the maximal anteroposterior distance of the medial (distance a) and lateral femoral (distance b) condyle and the minimal anteroposterior distance between the deepest point of the trochlear groove and the line paralleling the posterior outlines of the femoral condyles (distance c). Trochlear depth was calculated by the formula ([a + b]/2) − c.

Lateralization is the distance of the line paralleling the lateral margin of the lateral condyle to the most lateral point of the patella [21].

The four-group Dejour classification of trochlear dysplasia was used a guidance for evaluation of trochlear dysplasia, but due to the general reliability of this subjective classification, we preferred instead to base our indications on measurable MRI parameters [22].

The patella height was evaluated in a lateral knee radiograph. In patients with Caton-Deshamps [23] index > 1.2, a tibial tuberosity osteotomy with distalization was performed. In cases with a tibial tuberosity to posterior cruciate ligament distance > 20 mm, an additional medialization is considered; however, in none of the cases was this found to be indicated in this cohort. In cases when the femoral anteversion measured by the Waidelich method was ≥ 35 degrees, a distal de-rotational femoral osteotomy was performed [24].

### Subjective assessment

A standardized questionnaire including the following scores was administered to patients postoperatively: Marx Scale, Tegner Activity Scale, Kujala Score, and Banff II Score [25]. The Marx Scale and Tegner Scale are subjective questionnaires assessing activity levels. The Banff II and Kujala scores are specifically focusing on the patellofemoral pathology and were considered the primary outcome parameters. Both scores assess the subjective symptoms and functional limitations with patellofemoral disorders.

### Rehabilitation protocol

All patients had a postoperative rehabilitation following a standardized protocol at our institution:

For open or arthroscopic trochleoplasty + MPFL reconstruction, this rehabilitation was full weight bearing, using crutches until suture removal and with free range of motion.

For open or arthroscopic trochleoplasty + MPFL reconstruction with additional bony intervention, a brace with a range of motion limited to 70° of flexion and partial weight bearing (about 20–25 kg) is required for about 6 weeks.

Isometric quadriceps training and hamstring strengthening start at the day after surgery. Continuous passive motion (CPM) training is initiated on the first postoperative day.

Passive guided training is carried out with free range of motion. After 6 weeks weight bearing exercises are initiated.

Patients were discharged from the hospital when they reached 70° of flexion.

### Statistical analysis

Statistical analyses were performed using IBM SPSS Software Version 26 (IBM Corp., Armonk, NY, USA). Descriptive statistics including mean ± standard deviation were calculated. Parametric data were analyzed using the *t*-test. A value of *p* < 0.05 was considered significant.

### Ethics

The study protocol received approval from the Ethics Committee of the Federal State of Carinthia (A 43/18). Informed consent was obtained from all participants and/or their legal guardian(s). All procedures adhered to the principles outlined in the Declaration of Helsinki.

## Results

### Patients

**Fig. 4:**
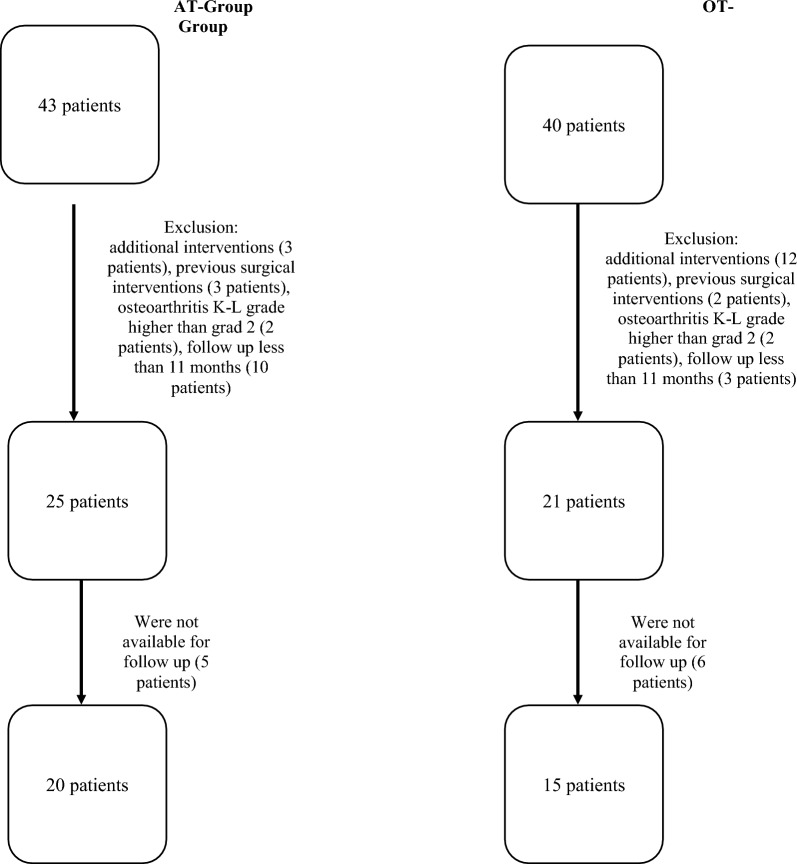
Flowchart for patient enrollment. *AT* arthroscopic trochleoplasty, *OT* open trochleoplasty, *K-L* Kellgren–Lawrence

In total, 15 patients (mean age 18.8 years ± 7.6; 12 female/3 male) were available for follow-up in the OT group, whereas in the AT group there were 20 (mean age 18.4 years ± 3.8; 6 female/14 male). The mean follow-up was 29.9 months ± 17.6 in the OT group and 12.7 months ± 1.6 in the AT group (Fig. 4).

### Clinical assessment

No postoperative dislocation was registered in either group. Preoperatively, all patients had a positive apprehension sign, and at follow-up all patients except two presented (in the OT group) a negative apprehension sign. The results of the subjective questionnaires show no significant difference between the two intervention groups (Table 1). In both groups, an improvement in all of our measured radiological results could be seen (Table 2).

**Table 1:** Evaluation subjective questionnaires at last follow-up

Variable	AT group (*n* = 20)	OT group (*n* = 15)	*p*-Value
Kujala Score	91.8 ± 6.8	91.4 ± 5.6	0.47
Banff II Score	90.6 ± 8.5	87.2 ± 8.7	0.78
Tegner Scale	3.3 ± 2.1	3.2 ± 0.9	0.01
Marx Scale	5.8 ± 6.0	2.8 ± 3.3	0.01

*AT* arthroscopic trochleoplasty, *OT* open trochleoplasty

**Table 2:** Radiological results at last follow-up

Variable	AT group (*n* = 20)	OT group (*n* = 15)	*p*-Value
LTI preop (degree)	7.7 ± 3.2	7.6 ± 3.9	0.41
LTI postop (degree)	23.9 ± 6.1	21.4 ± 5.5	0.51
Trochleadepth preop (mm)	2.3 ± 1	1.3 ± 1	0.36
Trochleadepth postop (mm)	7.2 ± 2	5.7 ± 2	0.49
Lateralization of the patella preop (mm)	11.1 ± 5	10.4 ± 6	0.73
Lateralization of the patella postop (mm)	2.0 ± 3	4.9 ± 4	0.62

*AT* arthroscopic trochleoplasty, *OT* open trochleoplasty, *LTI* lateral trochlea inclination, *postop* postoperatively, *preop* preoperatively

### Radiological results arthroscopic versus open technique

There was no significant difference in the postoperative radiological findings between both groups: lateral trochlear inclination [OT 21.4° (± 5.5°)/AT 23.9° (± 6.1°)], depth of the trochlear groove [OT 5.7 mm (± 2 mm)/AT 7.2 mm (± 2 mm)], and the patellar lateralization [OT 4.9 mm (± 4 mm)/AT 2.0 mm (± 3 mm)].

### Length of hospitalization

The length of hospitalization was on average 2 days longer in the open technique [OT 8.1 days (± 2.7 days)/AT 6.2 days (± 2.7 days); *p*-value 0.89].

### Surgical time

The surgical time (skin incision to closure/without additional surgery) was on average 19 min longer in the AT group [OT 141.3 min (± 24.8 min)/AT 160.4 min (± 35.3 min), *p*-value 0.08].

### Complications

One complications occurred in the OT group. One patient developed an intraarticular hematoma, which needed revision surgery. There were no major complications in the AT group.

### Second-look surgery

**Fig. 5:**
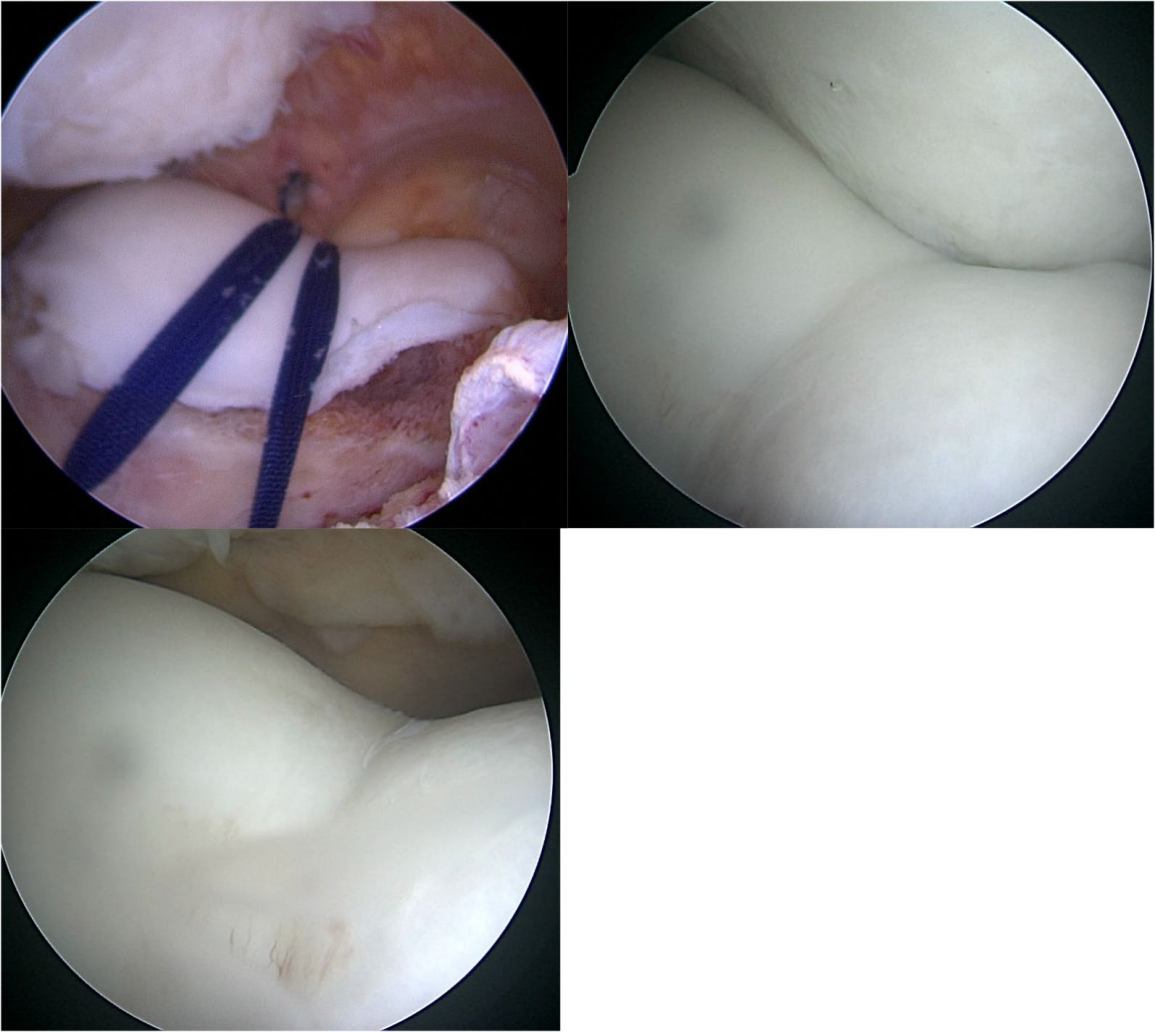
Removed subchondral bone has filled up with tissue (1 year after the primary surgery)

Diagnostic knee scope was performed 1 year after index surgery in three patients in the AT group. The previously removed subchondral bone has filled up with fibrotic tissue, and the inclination seemed to be improved compared with immediately after the trochleoplasty (Fig. 5).

## Discussion

The most important finding in the present study is that both open and arthroscopic technique have equivalent functional outcomes. Additionally, the radiological results achieved by the AT are comparable to those of the OT. A higher LTI was seen in the AT group; however, this was not statistically significant. The higher LTI might be the result of the better visualization due to the magnification of the arthroscope. The are no differences in the clinical outcome between the two technique. The results of the studied patient-reported outcome scores (PROMS) are comparable to the published data on open or arthroscopic technique [26].

The surgical objectives of the AT are consistent with those of the OT: the correction of a flat or nearly flat trochlea, to a minimum of inclination of < 17°; a central patella guidance; and unloading of the patellofemoral joint. Some studies have claimed that trochleoplasty causes increased contact pressure [27]. In this study the follow-up period was too short to observe or estimate any changes of the cartilage. No differences could be seen between the groups. Blond published a case report supporting the theory that trochlea dysplasia is associated with increased contact pressure and advocates for the deepening of the trochlea as a means of unloading [13].

However, pronounced patellofemoral osteoarthritis is seen in many untreated cases [28]. Further investigations regarding the condition of the cartilage after trochleoplasty are necessary. The results of this study indicate that both open and arthroscopic trochleoplasties are safe techniques, and this is in accordance with the systematic reviews from Leclerc et al. and from van Sambeeck et al. [29, 30]. In some of our cases, a diagnostic arthroscopy was done about 1 year after initial surgery. It could be observed that the removed subchondral bone had filled up with tissue and that the inclination improved compared with immediately after the trochleoplasty (Fig. 4). We hypothesize that this might be the consequence of the recentered patella and the additional contact pressure on the chondral flap.

Many studies deal with one surgical technique, but patella instability is multifactorial. The modern approach, as advocated by the Lyon group, of menu a la carte has become increasingly accepted.

We observed a longer surgical time in the AT group compared with the OT group. Part of the extended surgical time can be attributed to the application of a sterile tourniquet after completing the arthroscopic trochleoplasty procedure and before initiating the MPFL reconstruction. This preference for using a tourniquet during MPFL reconstructions contributes to the overall duration of the procedure. We found that the learning curve for the arthroscopic technique is similar to that of other arthroscopic techniques such as the anterior cruciate ligament (ACL) “all in-site” reconstruction [31]. As we have already accomplished, arthroscopic trochleoplasty will evolve to become simpler and more reproducible, particularly with advancements in arthroscopic techniques and equipment. However, in our opinion arthroscopic trochleaplasties should be performed by an experienced arthroscopic knee surgeon and with specific knowledge and understanding of trochlear dysplasia.

The postoperative sports activity level is low in both groups. However, we believe that implementing an extended rehabilitation protocol and a return to sport assessment could provide support for those patients who are motivated to improve (similar to the ACL reconstruction protocol). Mengis et al. reported about patients who were undergoing deepening trochleoplasty and medial soft tissue stabilization, with or without concomitant realignment surgery, achieving good clinical results and a high rate of return to sport participation. However, high-levels athletes could not reach their preoperative level [32, 33].

The major strength of this study is that it is the first clinical study that compares the radiological and clinical outcome of open versus arthroscopic trochleoplasty. To date, no such study has been published.

The study was limited by several factors: This study included a small number of cases and therefore may not be powered enough to rule out the possibility of a type II error. Furthermore, the study design is a retrospective analysis of prospectively collected data. Limitations included the heterogeneous distribution of study participants across groups, the short duration of the follow-up, and the differences in follow-up periods between the cohorts (12.7 months versus 29.9 months). However, the primary outcome parameters (radiological postoperative features on the MRI) from our study are not influenced by a longer follow-up.

## Conclusion

We found that patients undergoing an arthroscopic trochleoplasty for patella instability had a comparable outcome to those operated on using an open trochleoplasty. The findings of this study warrant further investigation in a prospective study with a long-term follow-up.

## Data Availability

The data that support the findings of this study are available from the corresponding author, upon reasonable request.

## Abbreviations

ACL: Anterior cruciate ligament
AT: Arthroscopic trochleoplasty
CPM: Continuous passive motion
CT: Computed tomography
LTI: Lateral trochlea inclination
MPFL: Medial patellofemoral ligament
MRI: Magnetic resonance imaging
OT: Open trochleoplasty
PROMS: Patient-reported outcome scores
TT-PCL: Tibial tuberosity to posterior cruciate ligament distance
